# A Lightweight Multi-Scale Context Detail Network for Efficient Target Detection in Resource-Constrained Environments

**DOI:** 10.3390/s25123800

**Published:** 2025-06-18

**Authors:** Kaipeng Wang, Guanglin He, Xinmin Li

**Affiliations:** Science and Technology on Electromechanical Dynamic Control Laboratory, Beijing Institute of Technology, Beijing 100081, China; 3120215105@bit.edu.cn (K.W.); 3120225110@bit.edu.cn (X.L.)

**Keywords:** target detection, lightweight neural network, multi-scale feature fusion, context-aware modulation, edge computing

## Abstract

Target detection in resource-constrained environments faces multiple challenges such as the use of camouflage, diverse target sizes, and harsh environmental conditions. Moreover, the need for solutions suitable for edge computing environments, which have limited computational resources, adds complexity to the task. To meet these challenges, we propose MSCDNet (Multi-Scale Context Detail Network), an innovative and lightweight architecture designed specifically for efficient target detection in such environments. MSCDNet integrates three key components: the Multi-Scale Fusion Module, which improves the representation of features at various target scales; the Context Merge Module, which enables adaptive feature integration across scales to handle a wide range of target conditions; and the Detail Enhance Module, which emphasizes preserving crucial edge and texture details for detecting camouflaged targets. Extensive evaluations highlight the effectiveness of MSCDNet, which achieves 40.1% mAP50-95, 86.1% precision, and 68.1% recall while maintaining a low computational load with only 2.22 M parameters and 6.0 G FLOPs. When compared to other models, MSCDNet outperforms YOLO-family variants by 1.9% in mAP50-95 and uses 14% fewer parameters. Additional generalization tests on VisDrone2019 and BDD100K further validate its robustness, with improvements of 1.1% in mAP50 on VisDrone and 1.2% in mAP50-95 on BDD100K over baseline models. These results affirm that MSCDNet is well suited for tactical deployment in scenarios with limited computational resources, where reliable target detection is paramount.

## 1. Introduction

Target detection in resource-constrained environments is a major challenge. Target detection in unmanned aerial vehicles (UAVs) faces significant challenges in complex urban or natural environments due to stringent constraints on computational power, memory, and real-time performance. These limitations make it impractical to deploy high-complexity models or load large neural network weights while excessive computational loads drastically reduce operational endurance. Consequently, conventional approaches often suffer from compromised accuracy and real-time detection performance. Lightweight target detection algorithms, characterized by low computational complexity and ease of deployment on edge devices, achieve an optimal balance between detection precision and real-time efficiency. Such algorithms are driving transformative applications in military reconnaissance, disaster search and rescue, smart healthcare, and traffic management, underscoring the critical importance of research in this field. Resource-constrained environments are ubiquitous across military, industrial, and civilian domains. By studying target detection algorithms in resource-constrained environments, we can provide great help for personnel search and rescue, traffic management and other civilian fields. As the representative of resource-constrained environments, the battlefield environment includes deserts, jungles, mountains, cities, oceans, etc. The diversified environment can provide good research support for us. By studying the military target recognition algorithm in the battlefield environment, its technology can be further applied to personnel search and rescue, traffic management, and other aspects. The battlefield environment is a complicated, informatization data space, and detection algorithms must be most incredibly responsive and specific, adapting to a continuously changing panorama for more and more intelligence accumulating and opportunity evaluation [1,2,3]. The development of efficient and robust target detection strategies has become a hot research direction in resource-constrained environments. This research is conducted exclusively for humanitarian and civilian applications, including search and rescue operations, traffic management, and disaster relief scenarios. The authors acknowledge the dual-use potential of object detection technology and emphasize that this work is intended to benefit civilian safety and emergency response capabilities.

The target detection algorithm in resource-constrained environments is constantly optimized. The first such approaches were based on segmentation techniques, which have separated possible targets from the background elements [4]. Fuzzy inference systems were later added to these to deal with environmental uncertainties and classification ambiguities [5]. Breakthroughs further took the form of Charge Coupled Device image processing for greater detection in variable lighting [6]. All the weather detection of submarines was obtained by fusing SAR data to early detection schemes, greatly improving capabilities [7,8].

Being applied as a target detection milestone, the ICA was a key milestone in the evolution of military target detection. The idea of the ICA was shown by Twari et al. [9], in whose work it was used on hyperspectral images to detect military targets, which provided better distinction between camouflaged objects and their surrounding environments. Consequently, spectrally modeled algorithms in conjunction with the ICA resulted in improvements in detection accuracy in a resource-constrained environment that is complex.

However, the deployment of such advancements in cluttered, unpredictable operational settings turned out to be a challenge for traditional methods. With high sensitivity to the variations in target appearance and orientation together with the environmental factors, they were dependent on the manually engineered features and thus could not be practically utilized in real resource-constrained scenarios.

The emergence of deep learning has revolutionized object detection methodologies, heralding a paradigm shift. Improving convolutional neural networks, like SqueezeNet [10], set the level for more effective detection frameworks. The following improvements in the area-based totally CNN (R-CNN) family, such as rapid R-CNN [11], Faster R-CNN [12], and Mask R-CNN [13], resolved some of the shortcomings from the early designs and made the manner for extra robust detection structures.

At the same time, single-stage detection frameworks and the Unmarried Shot MultiBox Detector (SSD) [14], as well as the YOLO family of detectors [15], appeared, simultaneously offering computationally efficient solutions that favored speedy detection at the expense of precision. The assignment of finding a balance between computational efficiency and precision, however, keeps forming research in the field.

Target detection in resource-constrained environments is extremely challenging with respect to conventional object detection. But objectives in resource-constrained environments are typically small on a sensor scale of view and are often partially obscured. Also, complex situations with changing climate, not to mention limited computational resources and need for real-time processing, make it intractable to develop powerful detection systems.

Despite such progress in civilian object detection using deep learning frameworks, their use in resource-constrained environments would be constrained. However, although a variety of conventional detection algorithms have been developed using general purpose approaches for general use, the performance of these algorithms in complex environments is not ideal. Two challenges remain: (1) algorithms need to adapt to complex and diverse environments and (2) the computational burden of the most advanced model exceeds the available resources for tactical problems, which brings great inconvenience to use.

This paper presents a novel approach to target detection in resource-constrained environments that specifically addresses these challenges through a hierarchical feature fusion architecture optimized for multi-scale, camouflaged target detection while maintaining computational efficiency. Our key contributions include the following:

1. A lightweight MSCDNet (Multi-Scale Context Detail Network) architecture that solves the computational resource constraints in resource-constrained environments.
2. A Multi-Scale Fusion (MSF) module that addresses the challenge of detecting targets with significant dimensional variations, camouflage, and partial occlusion.
3. A Context Merge Module (CMM) that overcomes the difficulty of integrating features from different scales for comprehensive target representation.
4. A Detail Enhance Module (DEM) that preserves critical edge and texture details essential for distinguishing camouflaged targets in complex environments.

The remainder of this paper is organized as follows: Section 2 reviews related work in object detection and military target recognition so as to provide reference for us to solve target recognition under limited resource environments in the civil field. Section 3 details our proposed methodology, including the YOLO11n architecture, Multi-Scale Fusion module, Context Merge Module, and Detail Enhance Module. Section 4 presents experimental results and comparative analysis. Section 5 concludes with a summary of findings and directions for future research.

## 2. Related Work

### 2.1. Traditional Military Target Detection Methods

Early military target detection relied on distinctive visual characteristics through edge and contour features. Sun et al. [16] proposed adaptive boosting for SAR automatic target recognition, demonstrating how ensemble learning could improve feature-based detection in radar imagery. The application of texture-based features such as Histogram of Oriented Gradients (HOG) [17] and Local Binary Patterns (LBP) improved discrimination between military targets and background elements. Zhang et al. [18] developed face detection based on multi-block LBP representation, a technique that was later adapted for military personnel detection. For non-rigid targets like soldiers, frameworks based on Deformable-Part Models (DPMs) offered flexibility in handling diverse postures and equipment configurations.

In sensor technology, Pei et al. [19] further explored multiview SAR automatic target recognition optimization, demonstrating how multiple perspectives can enhance detection reliability. Che et al. [20] developed multi-spectral fusion techniques combining infrared and visible light images. Addressing illumination variation issues, meanwhile, Li et al. [21] specifically researched target classification in low-light night vision conditions.

Motion characteristics of military targets also serve as important cues in traditional methods. Salmon et al. [22] studied the effects of motion on in-vehicle system operation in battle management systems, highlighting practical usability challenges in tactical environments. Bajracharya et al. [23] developed a fast stereo-based system for detecting and tracking pedestrians from moving vehicles, techniques later adapted for soldier tracking. Chaves [24] investigated Kalman filtering for low-cost GPS-based collision warning systems in vehicle convoys, demonstrating practical tracking applications.

Despite achieving success in specific controlled environments, these traditional methods faced numerous challenges: sensitivity to environmental changes, insufficient robustness against camouflaged targets, dependence on expert experience for feature engineering, and a lack of end-to-end learning capabilities. Boult et al. [25] addressed some of these limitations through specialized visual surveillance systems for non-cooperative and camouflaged targets in complex settings, but fundamental challenges remained that motivated researchers to transition toward deep learning methods.

### 2.2. General Deep Learning Methods for Military Target Detection

The emergence of deep learning, particularly Convolutional Neural Networks (CNNs), has fundamentally transformed military target detection research. Zhao et al. [26] applied the Faster R-CNN framework to storage tank detection using high-resolution aerial imagery, significantly improving detection accuracy. Peng et al. [27] utilized Faster R-CNN for multi-object extraction in complex backgrounds under artificial intelligence contexts, addressing background interference issues. Naz et al. [28] explored soldier detection using unattended acoustic and seismic sensors, optimizing detection systems for various battlefield conditions.

Single-stage detectors such as YOLO and SSD have also been widely applied to military scenarios due to their efficiency. Xu and Wu [29] improved the YOLOv3 model with DenseNet for multi-scale remote sensing target detection, achieving balance between speed and accuracy. Wang et al. [30] developed a lightweight detector based on SSD with depth-separable convolution, balancing computational efficiency and detection performance. These general object detection frameworks provide fundamental solutions for military target detection but require domain-specific adaptations to address unique military challenges.

With advancements in deep learning technology, emerging network architectures have been continuously applied to military target detection. Feature pyramid networks (FPNs) have been widely used to address scale variations in military targets. Wang et al. [31] developed a multi-scale infrared military target detection system based on the 3X-FPN feature fusion network, capable of processing targets at different scales. RetinaNet and its focal loss have been applied to address the foreground–background class imbalance in detection tasks. Liu et al. [32] improved RetinaNet for the high-precision detection of transmission line defects, effectively enhancing detection sensitivity.

The Transformer architecture has recently been introduced to military target detection with promising results. Pushkarenko and Zaslavskyi [33] researched areas in Ukraine affected by military actions using remote sensing data and deep learning architectures. Li et al. [34] developed a military target detection framework based on Swin Transformer, utilizing SwinF with feature fusion for enhanced target detection. These advanced architectures demonstrate superior performance in modeling long-range dependencies and contextual relationships, crucial for understanding complex battlefield scenarios.

To address specific requirements of military applications, researchers have developed specialized techniques. Zhuang et al. [35] proposed military target detection methods based on EfficientDet and Generative Adversarial Networks, simulating conditions such as smoke, dust, and partial occlusion. Sun et al. [36] introduced YOLO-E, a lightweight object detection algorithm specifically designed for military targets. Jani et al. [37] reviewed model compression methods for YOLOv5, addressing deployment constraints in resource-limited tactical environments. Zhang et al. [38] investigated multi-scale feature fusion networks for object detection in very high resolution optical remote sensing images, lowering deployment thresholds for field operations.

### 2.3. Deep Learning Methods for Specific Target Detection

The deep learning method can solve the unique characteristics of different targets and take tanks and soldiers as examples for analysis.

For tank detection, Fan et al. [39] developed a fast detection and reconstruction method for tank barrels based on component prior and deep neural networks in the terahertz regime, demonstrating how component-level detection can substantially improve performance. Ma et al. [40] proposed an end-to-end method with transformers for the 3-D detection of oil tanks from single SAR images, not only detecting overall position but also identifying key components, providing richer feature information for model recognition.

Addressing camouflage issues in target detection, Song et al. [41] developed a multi-granularity context perception network for the open set recognition of camouflaged objects, incorporating spatial and channel attention modules while utilizing contextual information to determine actual target contours against complex terrain backgrounds. Naeem et al. [42] explored multi-sensor fusion technologies, combining data with integrated multi-sensor data fusion algorithms that dynamically adjust sensor importance according to environmental conditions. Lv et al. [8] researched the recognition of deformation military targets in complex scenes via MiniSAR submeter images, highlighting the importance of integrating different sensor modalities.

Soldier detection presents different challenges due to non-rigid characteristics, multiple postures, and group behaviors. Wu and Zhou [43] designed video-based martial arts combat action recognition and position detection using deep learning, incorporating modules capable of adapting to morphological changes in different tactical postures. For camouflage detection, Choudhary [44] developed real-time pixelated camouflage texture generation techniques combining multi-scale texture descriptors to capture minute differences between camouflage equipment and natural environments. Barnawi et al. [45] provided a comprehensive review of landmine detection using deep learning techniques, enhancing detection through preprocessing techniques and designing feature extraction networks adapted to challenging environments.

Group behavior analysis for soldiers has also received significant attention. Anzer et al. [46] proposed frameworks incorporating semi-supervised graph neural networks that model spatial relationships for detecting tactical patterns. Wang et al. [47] developed free-walking pedestrian inertial navigation systems based on dual foot-mounted IMU, capable of understanding complex movements. These specialized approaches have significantly advanced target detection technology by addressing the unique challenges of each target type.

### 2.4. Key Challenges of Target Detection in Resource-Constrained Environments

Although target detection based on resource-constrained environments has made progress in deep learning, there are still some challenges. Camouflage and concealment reduce detection accuracy by up to 40% while scale variation and small target detection are obstacles, especially in aerial reconnaissance where targets occupy less than 1% of the image in question. Weather conditions like rain, fog, or snow can cause accuracy drops of 35–60%, and while multi-sensor fusion improves all-weather detection, it introduces synchronization and computational challenges. Deployment on edge devices with limited resources creates tension between model compression, energy efficiency, and inference speed. Additionally, limited training data and the complexity of overlapping targets reduce performance by 25–45%, especially in dense formations and dynamic scenarios requiring real-time processing.

To address these issues, we propose the MSCDNet architecture with specialized modules. The Multi-Scale Fusion Module (MSFM) tackles scale variation, the Context Merge Module (CMM) improves feature integration across scales, and the Detail Enhance Module (DEM) preserves critical details for detecting camouflaged or occluded targets. This hierarchical approach balances computational efficiency with enhanced detection performance, addressing the challenges of complex environments.

## 3. Methodology

### 3.1. Overview of Model

**Figure 1:**
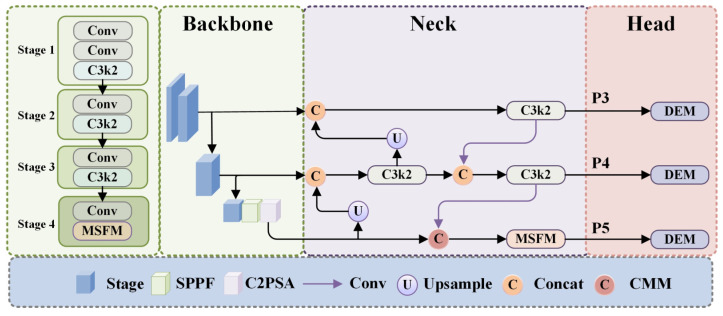
Overall structure diagram.

As illustrated in Figure 1, MSCDNet (Multi-Scale Context Detail Network) incorporates a Lightweight Perception Net toward lightweight network of efficient visual feature extract and object detection. The feature representation of this model is constructed gradually across multiple scales and yet guarantees computational efficiency on resource constrained environments. First, it is composed of some convolutional layers to set up some basic features and reduce spatial dimensions. C3k2 [48] modules, with a 2 × 2 kernel and bottleneck design that process these features, are optimized for computational efficiency and representation capacity. The architecture then incorporates three key components for target detection: Multi-Scale Fusion (MSF) modules merge information from diverse receptive fields to capture the multi-scale nature of targets, the Spatial Pyramid Pooling—Fast (SPPF) module aggregates multi-scale contextual information efficiently, and the C2PSA (Cross-Stage Partial with Position-Sensitive Attention) modules refine features with attention mechanisms that highlight relevant information while reducing background noise, improving detection precision.

The architecture implements a feature pyramid network design that combines features across multiple scales through upsampling operations and Concat modules in the detection head. Unlike data augmentation techniques that increase dataset diversity during preprocessing, upsampling here is an integral part of the network’s structure that restores the spatial resolution of higher-level feature maps (with strong semantic information) to match that of lower-level features (with fine-grained details). As context-aware mechanism and adaptive feature modulation strategies, the Context Merge Module (CMM) modules replace the simple concatenation approach with a more powerful way of cross-scale feature integration by making use of the complementary information provided by multiple feature levels. C3k2 modules will further amplify the feature’s discriminative power after being refined at each scale of the feature pyramid. Finally, these multi-scale features pass through the Detail Enhance Module (DEM) that processes them to the final predictions while keeping critical details, hence being especially important for military target identification.

The perception network obtained in this modular design is a construction, refinement, and integration of visual features at different scales. The network is composed of each specialized module, which helps detect objects of different sizes, and is computationally efficient. Through this seamless coordination, MSCDNet can generate superior detection performance in the presence of complex environments with targets of intricate morphologies, high background interference, and diverse scale whilst maintaining a lightweight and suitable for constrained resource edge deployments.

### 3.2. Multi-Scale Fusion Modulation

**Figure 2:**
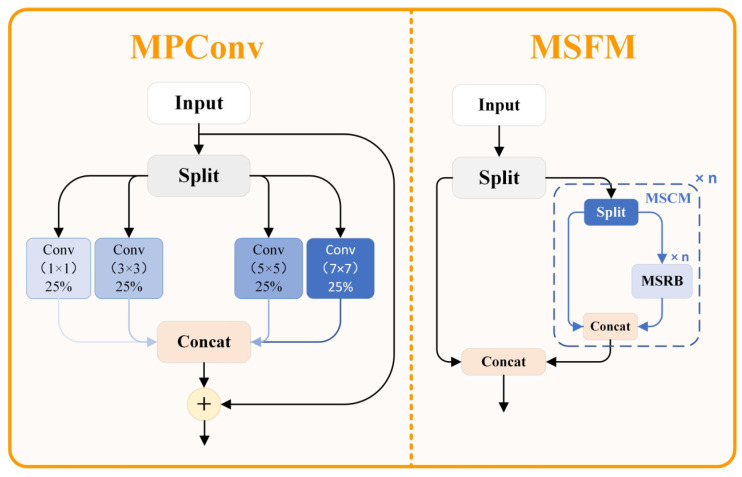
MSFM structure diagram.

The use of traditional object detection networks proves difficult when applied to resource-constrained settings. The detection targets include objects of various sizes extending from tiny ones at distance to large equipment near the scene, therefore leading to substantial scale variability. The position and shape of the target in the complex environments also have great changes. The detection requires superior algorithms that can extract features efficiently. Basic features extraction techniques succeed during their intended operation though they collapse at capturing multi-scale details and retaining spatial data especially within challenging environments. The MSFM (Multi-Scale Feature Modulation) framework serves as our proposed detection enhancement solution by applying multi-scale convolution strategies and effective feature union approaches in a structure demonstrated in Figure 2.

The efficient multi-scale convolution modules within the MSFM structure function to improve feature representation quality. Partial feature separation together with advanced multi-scale convolution strategies enables this framework to improve its capability for targets with different scales. The MPConv (Multi-scale Parallel Convolution) module stands as the central aspect of the MSFM by maximizing input information from different receptive fields through parallel multi-scale convolution together with advanced feature reorganization methods.

The MPConv module is the cornerstone innovation of the MSFM structure, and its mathematical formulation is outlined in Equation (1):

(1) $$F_{out}=\phi \left(\sum_{i=1}^{n}\omega_{i}\cdot \left(W_{i}*X_{i}\right)+\beta \cdot \sqrt{\prod_{i=1}^{n}∥W_{i}*X_{i}{∥}_{F}}\right)\cdot \gamma$$

The specific mathematical implementation of the MPConv module can be further decomposed. Initially, the input feature X is uniformly divided into n groups along the channel dimension, with each feature group containing C/n channels when the input feature X has C channels. Subsequently, each feature group is processed through convolution kernels of different sizes, with typical kernel size configurations being $\left[1\times 1,3\times 3,5\times 5,7\times 7\right]$, enabling the model to simultaneously capture spatial information at different scales. The processed features from each group are then reconnected along the channel dimension, and finally, feature fusion and channel dimension adjustment are accomplished through a 1×1 convolution to obtain the final output feature.

The MPConv module functions through a Fourier analysis method that performs multi-scale frequency selection filtering. Organizational structure of convolutional filters includes large and small kernels that extract different frequency ranges where the sizes of convolutional filters determine the range of information acquired with large filters maintaining contours and big features whereas small ones capture edges and tiny details. The sequence of parallel multi-scale filtering operations maintains plentiful frequency content without eliminating high-frequency details as traditional convolution frameworks would typically do. The frequency response of the filters can be represented via Fourier transformation, as shown in Equation (2):

(2) $$H_{i}\left(u,v\right)=F\left(W_{i}\right)\left(u,v\right)\cdot e^{j\theta_{i}\left(u,v\right)}\cdot \Lambda_{i}\left(u,v\right)$$

Here, F denotes the Fourier transform operator, $H_{i}\left(u,v\right)$ represents the frequency response of the i-th convolution kernel, $\theta_{i}\left(u,v\right)$ is the phase response function, and $\Lambda_{i}\left(u,v\right)$ is the filter characteristic curve. The comprehensive module’s frequency response is the synthesis of individual components, as shown in Equation (3):

(3) $$H\left(u,v\right)=\sum_{i=1}^{n}\alpha_{i}\left(u,v\right)\cdot H_{i}\left(u,v\right)\cdot F\left(X_{i}\right)\left(u,v\right)+\Omega \left(u,v\right)\cdot \sqrt{\prod_{i=1}^{n}{\left|H_{i}\left(u,v\right)\cdot F\left(X_{i}\right)\left(u,v\right)\right|}^{2}}$$

In the MSFM implementation, the MPConv module is embedded into a basic residual unit, forming a Multi-Scale Residual Block (MSRB) as shown in Equation (4):

(4) $$F_{MSRB}=X+\mathrm{MPConv}\left({\mathrm{Conv}}_{1\times 1}\left(X\right)\right)$$

Here, the initial 1 × 1 convolution operation compresses the input channels to c (typically half of the output channels), enabling efficient cross-channel information fusion and dimensionality reduction without spatial information loss. This operation significantly reduces the computational complexity of subsequent multi-scale feature extraction while maintaining representational capacity. The MPConv operation then implements multi-scale feature extraction, and finally, the original features are added to the processed features through a residual connection. This residual connection mechanism not only mitigates the vanishing gradient problem in deep networks but also achieves effective fusion of features at different abstraction levels.

The Multi-Scale Dual-path Module (MSDM) represents a dual-path feature extraction structure that replaces basic units with MSRBs as shown in Equation (5):

(5) $$F_{MSDM}=Concat\left(X_{path1},Sequential\left(\{{\mathrm{MSRB}}_{i}{\}}_{i=1}^{n}\circ \Psi \left(\theta \right)\right)\left(X_{path2}\right)\right)\cdot \sqrt{\eta \cdot Norm\left(X_{path1}\right)\cdot Norm\left(X_{path2}\right)}$$

In this formulation, the input feature X is divided into two parts, $X_{path1}$ and $X_{path2}$. $X_{path1}$ is directly transmitted to the output while $X_{path2}$ is processed through n serially connected MSRB modules before being transmitted to the output. This dual-path design balances the depth and width of feature extraction, enabling simultaneous preservation of low-level detail features and high-level semantic features.

Ultimately, MSFM employs MSDM as its fundamental building unit, as shown in Equation (6):

(6) $$F_{MSFM}=\mathrm{Concat}\left(X_{path1},\mathrm{ModuleList}\left({\mathrm{MSDM}}_{1},{\mathrm{MSDM}}_{2},...,{\mathrm{MSDM}}_{n}\right)\left(X_{path2}\right)\right)$$

This multi-level feature extraction and fusion mechanism can capture rich feature information across various scales, being particularly suitable for processing targets with complex morphologies and variable scales in resource-constrained scenarios.

From an information theory perspective, MSFM reduces information loss during feature extraction through parallel multi-scale processing, increasing the mutual information between input features and target features. It maximizes the mutual information as shown in Equation (7):

(7) $$I\left(X;Y\right)=H\left(Y\right)-H\left(Y|X\right)+\Delta \left(\kappa \right)\cdot D_{KL}\left(p\left(Y|X\right)∥q\left(Y\right)\right)-\lambda \cdot Tr\left(\Sigma_{XY}\Sigma_{YX}\right)$$

Here, *H(y)* represents the entropy of target features measuring information uncertainty, H(y|x) denotes the conditional entropy of target features given input features indicating remaining uncertainty, *I(x)* is an information gain function quantifying feature importance, *KL* is the Kullback–Leibler divergence metric measuring distribution differences, and *Tr*($\Sigma_{XY}\Sigma_{YX}$) represents the trace operation on feature covariance matrices that evaluates feature–background separability.

Instead of traditional multi-scale feature extraction methods, MSFM has several key advantages. MPConv is a parallel module that processes convolutions at various scales in parallel, thus maintaining the spatial information across scales and keeping away from the information loss in cascaded convolutions occurring with conventional convolutions. The second contribution is that the channel grouping strategy reduces computational complexity, but maintains satisfactory feature extraction capability, and has orders-of-magnitude-lower computational complexity than typical multi-branch structures. Third, MSFM handles the vanishing gradient problem of deep networks when using residual connections for the feature fusion of low- and high-level features. The efficient use of multi-scale convolution in the MSFM structure enables significant improvements in feature representation over multi-scale target identification in resource-constrained detection scenarios.

### 3.3. Context Merge Module

**Figure 3:**
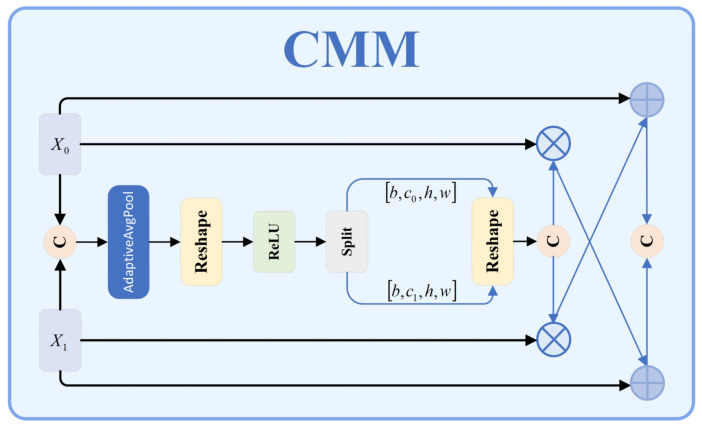
CMM structure diagram.

Traditional object detection networks simply concatenate or fuse the feature by summing the features. Nevertheless, the performance in target detection is poor because many specific targets have complicated shapes, different scales, and strong interference of the background. Conventional fusion techniques do not fully capture complementary information at the different feature levels; hence, they sacrifice efficiency in fusion and target feature capture. The issue of concatenation operations is that they simply add features of different levels without considering contextual correlations or adapting to their own features, which leads to reductions in detection accuracy. We propose the Context Merge Module (CMM) as shown in Figure 3, which involves context-aware mechanisms and adaptive modulation strategies to improve the feature extraction and to achieve higher accuracy and robustness in specific target recognition.

The key idea of the CMM design is to leverage contextual information to enable the adaptive fusion of features at varying levels to achieve synergy between different levels’ features, making the most of the complementary feature relations to improve the model’s feature roughness. In the module, there are four key components: feature adjustment layer, feature concatenation operation, channel attention mechanism, and complementary feature fusion. The CMM module first makes a feature adjustment layer to make the channel dimension compatible between features at different levels. When the number of channels in input feature x₀ differs from that in feature x_1_, a 1 × 1 convolution operation is applied to feature x_0_ to adjust its channel count to match that of feature $x_{1}$.

Second, the adjusted feature $x{’}_{0}$ is concatenated with feature $x_{1}$ along the channel dimension to form a feature concatenation vector $x_{\mathrm{concat}}$.

Third, the concatenated feature is processed through a Squeeze-and-Excitation (SE) attention module, which first performs global average pooling on the feature to capture global contextual information between channels, then learns the correlations between channels through a non-linear transformation constructed by two fully connected layers, ultimately generating channel attention weights, as shown in Equation (8):

(8) $$W=\sigma \left(W_{2}\cdot \delta \left(W_{1}\cdot \mathrm{GAP}\left(x_{\mathrm{concat}}\right)+b_{1}\right)+b_{2}\right)\cdot \alpha +\beta \cdot tanh\left(\gamma \cdot \mathrm{GAP}\left(x_{\mathrm{concat}}\right)\right)$$

Here, GAP denotes the global average pooling operation; $W_{1}$ and $W_{2}$ represent the weight matrices of the respective fully connected layers; $b_{1}$ and $b_{2}$ denote the corresponding bias terms; δ signifies the ReLU activation function; σ represents the Sigmoid activation function, while α, β, and γ are learnable scaling parameters; and the tanh function provides additional non-linearity to enhance expressiveness.

The generated channel attention weights W are subsequently bifurcated into two segments corresponding to weights for $x{’}_{0}$ and $x_{1}$, respectively. These weights are then applied to the original features through element-wise multiplication to derive weighted feature representations.

Finally, through a cross-enhancement feature strategy, the weighted features and original features undergo complementary fusion, as expressed in Equation (9):

(9) $$x_{\mathrm{out}}=Concat\left(\lambda_{1}\cdot \left(x{’}_{0}+\Phi \left(x_{1_{\mathrm{weighted}}},\theta_{1}\right)\right)⊕\lambda_{2}\cdot \left(x_{1}+\Psi \left(x_{0_{\mathrm{weighted}}},\theta_{2}\right)\right)\right)\cdot \sqrt{\eta \cdot Norm\left(x{’}_{0}\right)\cdot Norm\left(x_{1}\right)}$$

Here, Φ and Ψ represent non-linear transformation functions with parameters $\theta_{1}$ and $\theta_{2}$, respectively; $\lambda_{1}$ and $\lambda_{2}$ are balancing factors; ⊕ denotes channel-wise concatenation; Norm signifies feature normalization; and η is a global scaling factor.

By preserving the original features and subsequently integrating context-enhanced information from other features, the Context Merge Module (CMM) helps the feature fusion remain efficient by preserving complementarity. Within the enhanced YOLO11 network, the CMM is strategically made to replace traditional feature concatenation as part of the multi-scale feature fusion. It is used at critical stages (from higher-level to lower-level features, P_5_→P_4_, P_4_→P_3_; from lower-level (P_3_) to higher-level (P_4_, P_4_) features). Optimizing feature data to each scale of the environment, this multi-tiered approach is an improvement to the detection of military targets at multiple sizes. The CMM retains diverse feature characteristics by combining channel attention with complementary fusion, adapting to them to weight and integrate more precisely. Finally, cross-enhancement further improves the accuracy of detection of specific targets like small and multi-scale targets in cluttered environments. Experiment results demonstrate the advantage of using YOLO11 network over the CMM for specific target detection. CMM’s principles also suggest ways of dealing with feature fusion issues in other computer vision areas.

### 3.4. Detail Enhance Module

**Figure 4:**
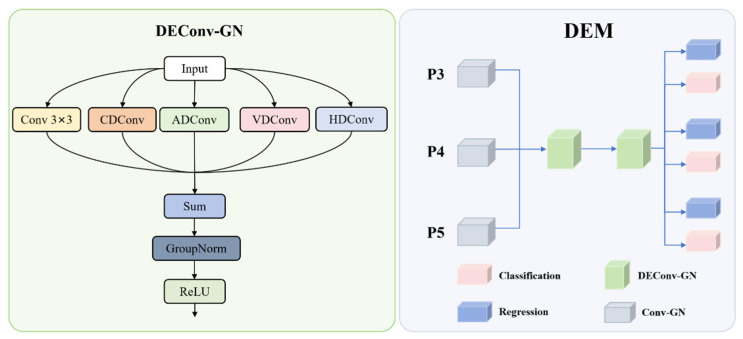
DEM structure diagram.

The Detail Enhance Module is a step up from object detection technology, mainly with regards to applications such as tank and soldier detection. Based on strong capabilities in advanced detection frameworks, this detection head incorporates the modifications that help feature representation and detection precision, especially for difficult targets. DEM architecture represents a novel integration of grouped normalization and detail-enhanced convolutions, being distinctly different from existing parallel convolution approaches. While conventional methods typically use uniform parallel branches, our DEM introduces specialized directional convolutions (CD, HD, VD, AD, STD) that capture specific geometric features, creating a unique multi-directional feature enhancement strategy not found in prior work. The innovation resides in the shared convolutional structure, which is trained to capture the details at the boundaries that are imperative for precise identification of specific targets against clutter and challenging environments. The DEM structure diagram is shown in Figure 4.

The DEM architecture centers around the Detail-Enhanced Convolution (DEConv-GN) module, which enhances edge and texture details. The DEConv architecture employs five specialized convolution branches, each designed for specific feature enhancement purposes: CD (Central Difference) convolution emphasizes central pixel variations and fine-grained texture details crucial for distinguishing camouflaged targets from background; HD (Horizontal Difference) convolution captures horizontal edge features that are essential for detecting object boundaries and structural elements; VD (Vertical Difference) convolution detects vertical edge features that complement horizontal information for complete boundary detection; AD (All Direction) convolution captures diagonal and multi-directional features that standard convolutions might miss; STD (Standard) convolution provides baseline 3 × 3 feature extraction as a reference branch. These paths combine through a Sum operation, followed by GroupNorm and ReLU activation. In DEM, DEConv-GN replaces batch normalization with group normalization to improve stability across varying batch sizes and conditions typical in surveillance. The detection head processes multi-scale features (P3, P4, P5) through initial Conv-GN layers, followed by a shared detail-enhanced convolution module with two DEConv-GN layers. This module ensures efficient cross-scale feature learning, after which the features are split for regression and classification tasks.

Mathematically, the feature transformation process reduces the input feature map dimensions, effectively compressing channel dimensions while preserving discriminative information necessary for accurate specific target detection. Detail enhancement occurs in the shared module as shown in Equation (10):

(10) $$F_{i}^{\prime \prime }={DEConvGN}_{2}({DEConvGN}_{1}(F_{i}^{\prime }))$$

The DEConv operations can be collectively expressed as shown in Equation (11):

(11) $$\mathrm{DEConv}\left(x\right)=Act\left(GN32\left(\sum_{j\in \{cd,hd,vd,ad,std\}}\omega_{j}\cdot {\mathrm{Conv}}_{j}\left(x\right)+\gamma \cdot \sqrt{\prod_{j\in \{cd,hd,vd,ad,std\}}∥{\mathrm{Conv}}_{j}\left(x\right){∥}_{2}}\right)\right)$$

Here, $\omega_{j}$ represents adaptive weights for each convolution branch learned during training, γ denotes the cross-branch interaction coefficient, $∥{\mathrm{Conv}}_{j}\left(x\right){∥}_{2}$ calculates the L2-norm of convolution outputs to represent feature significance, GN32 implements the Group normalization function with 32 groups, and Act applies a non-linear activation function such as ReLU. Group normalization function has 32 groups, a configuration that provides optimal balance between normalization effectiveness and computational overhead based on established guidelines from the original GN paper.

For efficient deployment, the DEConv module can be simplified by fusing the parallel convolution branches into a single convolution operation as shown in Equations (12) and (13):

(12) $$W_{\mathrm{fused}}=\sum_{j\in \{\mathrm{cd},\mathrm{hd},\mathrm{vd},\mathrm{ad},\mathrm{std}\}}\lambda_{j}\cdot W_{j}\cdot \Phi_{j}\left(\Theta_{j}\right)$$

(13) $$b_{\mathrm{fused}}=\sum_{j\in \{\mathrm{cd},\mathrm{hd},\mathrm{vd},\mathrm{ad},\mathrm{std}\}}\mu_{j}\cdot b_{j}\cdot \Psi_{j}\left(\Omega_{j}\right)$$

Here, $W_{j}$ represents convolution weight matrices for each branch, $b_{j}$ denotes the bias vectors for each branch, $\lambda_{j}$ and $\mu_{j}$ are branch importance coefficients determined during the optimization phase, and $\Phi_{j}\left(\Theta_{j}\right)$ and $\Psi_{j}\left(\Omega_{j}\right)$ implement transformation functions with learnable parameters $\Theta_{j}$ and $\Omega_{j}$ that enable adaptive branch fusion during model compression.

Following the shared feature processing, the detection head splits into two parallel branches: a regression branch that predicts the bounding box coordinates through a distribution focal loss (DFL) formulation and a classification branch that predicts class probabilities for targets. The regression output is processed as shown in Equation (14):

(14) $$B_{i}={\mathrm{Scale}}_{i}\left({\mathrm{Conv}}_{1\times 1}\left(F_{i}^{\prime \prime },4\times {\mathrm{reg}}_{\max}\right)\right)\cdot \sqrt{\eta \cdot Norm\left(F_{i}^{\prime \prime }\right)}\cdot \Gamma \left(\kappa \right)$$

Here, ${\mathrm{Scale}}_{i}$ represents a scale-specific adjustment factor for different feature levels, ${\mathrm{reg}}_{\max}$ denotes the number of bins for distribution focal loss, η is the feature normalization coefficient, and $\Gamma \left(\kappa \right)$ implements an adaptive scaling function with parameter κ controlling detection confidence

The classification process applies specialized weight matrices to the encoded feature representations, mapping them to appropriate class probability distributions and confidence scores for effective object categorization. The DFL methodology partitions each bounding box coordinate into ${\mathrm{reg}}_{\max}$ discrete bins, facilitating more precise localization of specific targets. During inference, the discrete probability distribution is converted to continuous coordinate values as expressed in Equation (15):

(15) $$b_{\mathrm{continuous}}=\sum_{j=0}^{{\mathrm{reg}}_{\max}-1}P\left(b_{j}\right)\cdot j+\delta \cdot \sum_{j=0}^{{\mathrm{reg}}_{\max}-1}P\left(b_{j}\right)\cdot log\left(j+\epsilon \right)\cdot e^{\tanh\left(\rho \cdot j\right)}$$

Here, $P\left(b_{j}\right)$ represents the probability of the coordinate value falling within bin j, δ is the distribution refinement parameter, ϵ denotes a small constant ensuring numerical stability, and ρ serves as the bin importance modulation factor that enables sub-bin precision beyond the discrete quantization level. Comprehensive detection output integrates both classification and localization branches, combining class probability scores with spatial position information to generate complete detection results. Predicted box distributions undergo decoding into actual spatial coordinates utilizing anchor references and stride scaling factors as formulated in Equation (16):

(16) bbox=dist2bbox(DFL(B),anchors)×strides

Applying the new DEM architecture approach is a step to solve the target detection challenge in a resource-constrained environment with advanced convolution techniques, as traditional approaches sometimes disregard the useful textural and edge details. It allows for fast inference time for real-time deployment because its parameter sharing is efficient and strong detection capabilities are maintained while reducing model size. Due to its good performance in detecting small objects, which are essential for long-range search and rescue, and early target identification, this architecture is favorable. DEM’s stable performance under various lighting and camouflage conditions is ensured by group normalization, and field tests confirm it to be superior in detecting camouflaged targets and at detecting distant personnel. It performs computationally efficiently in timing and accuracy, in the context of search and rescue operations, to support timely and accurate information and to optimize resource use.

## 4. Experiments

### 4.1. Experimental Details and Evaluation Criteria

These experiments were conducted on a system running Windows 10, with Python 3.10.16 and PyTorch 2.3.0. The hardware setup featured an RTX 3080 GPU, an Intel i7-11700K CPU, and CUDA version 11.8. For training, an SGD optimizer was used with a learning rate of 0.01 and a batch size of 32.

In object detection tasks, there are common performance metrics such as precision, recall, average precision (AP), and mean average precision (mAP). Osmosis is used to evaluate accuracy by precision, i.e., the ratio of correctly identified objects from all detected objects. Sensitivity (recall) is defined as proportion of positive samples that have been correctly identified and is only a fraction of positive samples.

Often these two metrics show inverse relationship between them. Higher recall means that the model is indeed finding most of the true positives, but the precision will decrease because of false positives. High precision means that the model is likely to make correct predictions; however, high precision also means that some objects may be missing; hence, precision falls.

The Equations (17)–(20) for calculating recall, precision, AP, and mAP are as follows:

(17) $$Recall=\frac{TP}{TP+FN}$$

(18) $$Precision=\frac{TP}{TP+FP}$$

(19) $$AP=\int_{0}^{1}P\left(r\right)dr$$

(20) $$mAP=\frac{1}{n+1}AP_{i}$$

### 4.2. Datasets

**Figure 5:**
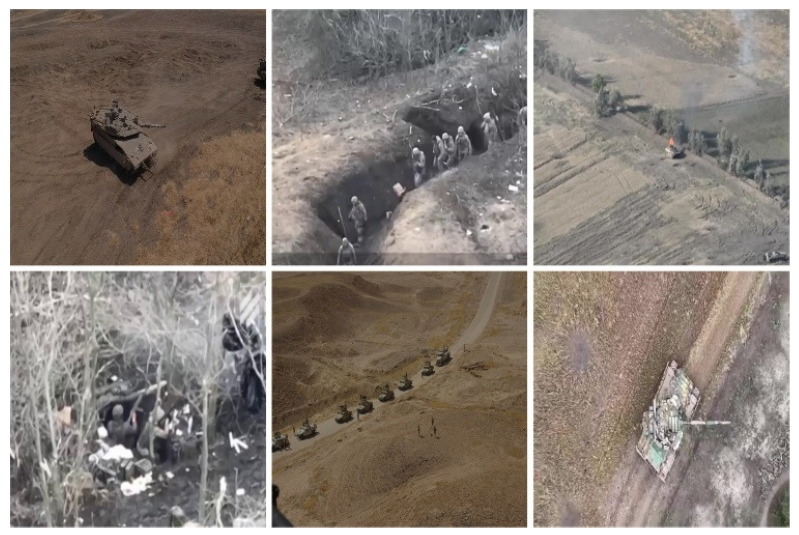
An example of dataset.

**Figure 6:**
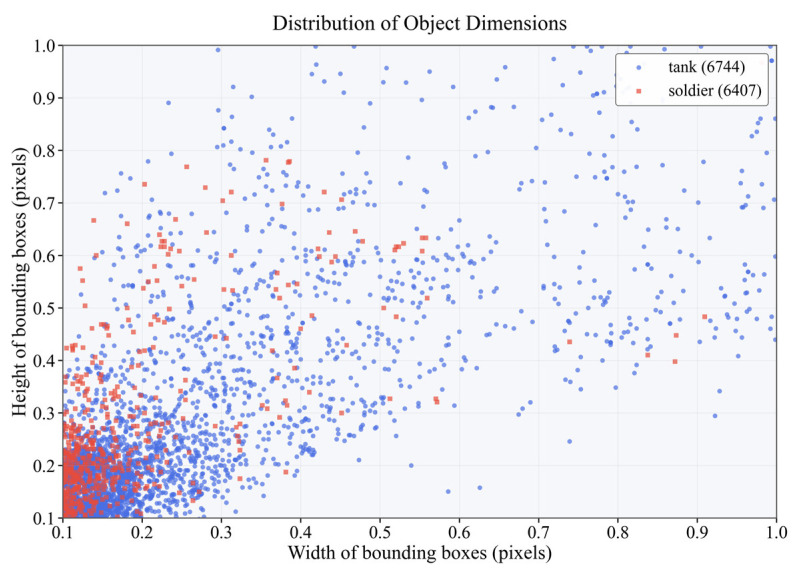
The distribution path of each category of the dataset.

In order to carry out research on efficient target detection in resource-constrained environments, we present a comprehensive dataset of 4616 images carefully chosen to enable solely tank and soldier detection in intricate environments as seen in Figure 5 and Figure 6. Then, we present a dataset that consists of real-world footage from the Russia–Ukraine war and high-quality military simulation images that are close to real battlefields but with controlled variation.

To create a challenging dataset, we have intentionally designed the dataset to include such detection scenarios as multi-scale targets, terrain occlusion, environmental obscurants (smoke and fog), advanced camouflage, and image degradation (which is typical in reconnaissance videos). The dataset concentrates on aerial views obtained from unmanned aerial vehicles (UAVs) and complemented by ground-level perspectives, corresponding to the visual difficulties in performing modern complex environments.

It is divided into 3 parts: 3231 images for training, 924 images for testing, and 461 images for validation. The purpose of this carefully assembled set is to serve as the basis for the creation of reliable detection algorithms to work in the harsh visual environment, resulting in better performance of the automated target detection systems.

### 4.3. Ablation Study

This ablation study evaluated the contributions of three key modules in MSCDNet: the Multi-Scale Fusion Model (MSFM) as shown in Table 1, the Context Merge Module (CMM), and the Detail Enhance Module (DEM). The baseline model, with 2.58 M parameters and 6.3 G FLOPs, achieved an mAP50-95 of 38.2%. MSFM improved precision by 3.1% and mAP50-95 by 0.9% while reducing parameters by 0.05 M. CMM increased recall by 1.3% and mAP50-95 by 0.4%. DEM reduced parameters by 0.32 M, decreased FLOPs by 0.3 G, and increased mAP50-95 by 1.4%. The combinations of MSFM-CMM, MSFM-DEM, and CMM-DEM demonstrated further improvements: MSFM-CMM raised mAP50-95 by 1.3% and precision by 3.6%; MSFM-DEM achieved a 0.7% increase in mAP50-95 and reduced parameters by 0.32M; CMM-DEM reached 39.6% mAP50-95 with reduced computational demands.

The complete architecture incorporating all three modules achieved superior performance with precision increasing to 86.1%, recall improving to 68.1%, and mAP50-95 reaching 40.1% while utilizing only 2.22 M parameters and 6.0 G FLOPs. These results validate our design approach, showing that MSF enhances feature representation, CMM improves cross-modal information use, and DEM optimizes efficiency without sacrificing accuracy.

**Figure 7:**
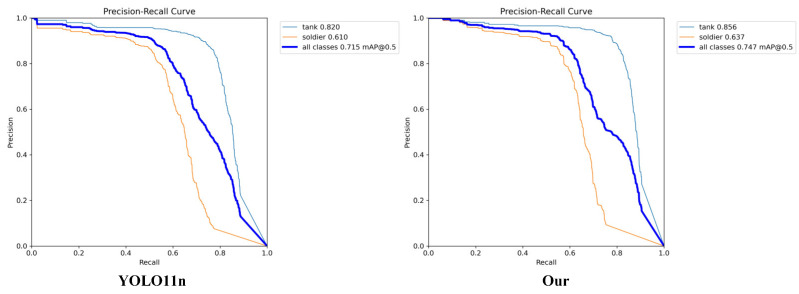
Comparison of PR curves before and after improvement.

The PR curve comparison in Figure 7 demonstrates the improved detection performance of our enhanced model. The curve for the improved model consistently maintained higher-precision values across varying recall levels, particularly in the mid-to-high recall range (0.5–0.8), where the improved model showed significant advantage over the baseline. This indicates better confidence in predictions and fewer false positives while maintaining high recall, reflecting the synergistic benefits of the three modules working together.

**Figure 8:**
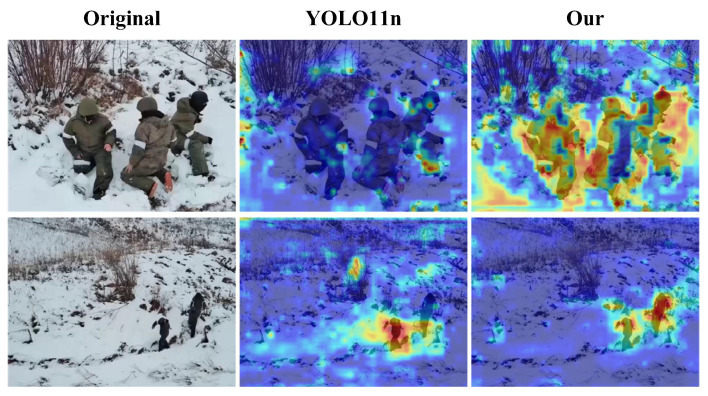
Comparison of the CAM effects of the model before and after the improvement.

The CAM (Class Activation Map) visualization in Figure 8 reveals the attention mechanism differences between the baseline and improved models. In Figure 8, the first column displays the original images, the second column presents the detection results from the YOLO11n model, and the third column shows the detection results from our proposed model. The improved model demonstrated more focused and precise activation regions that closely aligned with the actual target objects, particularly highlighting discriminative features rather than background elements. This enhanced attention localization explains precision improvements as the model more accurately concentrated on relevant target features while effectively suppressing background interference, a direct result of the MSF’s improved feature representation and CMM’s enhanced cross-modal information integration.

### 4.4. Comparison with State of the Art

MSCDNet demonstrates an exceptional efficiency-accuracy balance compared to contemporary object detection models, as shown in Table 2. Traditional architectures like SSD showed modest performance, with mAP50-95 8.3% lower than our model while requiring over 5 times more computational resources. More advanced models like DETR [49] and TOOD [50] offered improved accuracy but demanded substantially higher computational resources, with TOOD requiring 33 times more FLOPs than our approach.

Recently, lightweight models have provided more relevant comparisons. Our architecture outperformed RTMDet-Tiny [51] by 2.8% in mAP50-95 while using 25% fewer FLOPs and 54% fewer parameters. Similarly, it exceeded DFINE-n [52] and DEIM-n [53] variants by 2.6% in detection accuracy while reducing computational demands by 15% and parameter count by 40%.

The YOLO family shows progressive improvements across generations, yet our MSCDNet still demonstrates clear advantages. Compared to YOLOv5n [54], our model achieved 2.5% higher mAP50-95 with only slightly increased computational cost. Against YOLOv8n [55], we delivered a 2.3% accuracy improvement while reducing FLOPs by 26% and parameters by 26%. Compared to YOLOv10n [56], the closest competitor, our architecture improved mAP50-95 by 1.8% while reducing both parameter count and computational demands by nearly 27%. Our model also outperformed YOLOv11n by 1.9% in accuracy while using 4.8% fewer FLOPs and 14% fewer parameters.

Most notably, MSCDNet achieved balanced improvements across both precision and recall metrics, with values 4.3% and 1.6% higher than those of YOLOv11n, respectively, demonstrating superior detection capability across diverse object classes and challenging scenarios.

**Figure 9:**
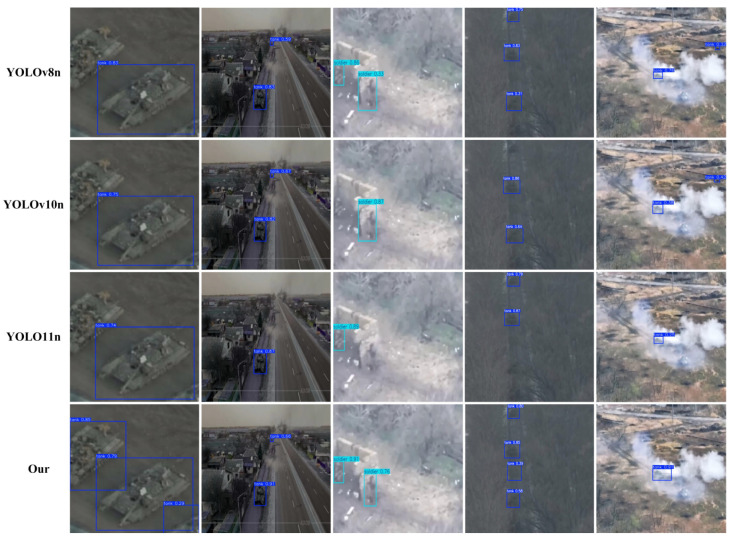
Detection effects of different models.

As shown in Figure 9, we conducted visual detection comparisons across different models. According to the detailed visual comparison analysis of the detection results, MSCDNet showed better detection performance than the comparison method in the complex scenarios. In the first column of aerial scenes, YOLOv8n, YOLOv10n, and YOLOv11n all had obvious missing detection problems, and could only detect a single main target in the scene, while MSCDNet not only detected the main large target but also successfully identified the partially occluded targets that were completely missed by other methods, showing stronger adaptability to complex scenes. In the second column of urban road aerial photography, YOLOv11n showed serious missed detection, only one target was detected and the confidence level was low, MSCDNet achieved complete object detection coverage, and the confidence level of all detected targets remained at a high level, indicating that the accuracy of feature extraction and target localization was better. In the smoke environment scene in the third column, in the face of the challenging conditions of low visibility, both YOLOv10n and YOLOv11n had missed detection, and could only identify one target, while MSCDNet and YOLOv8n detected all targets. However, MSCDNet demonstrated superior localization capability with more balanced and stable confidence distribution, verifying its robustness in maintaining detection performance and effectively preserving detailed information under harsh visual conditions, fully reflecting the technical advantages of its lightweight architecture design in sustaining high detection accuracy. In the fourth column (low-light conditions), all five tanks were partially missed by YOLOv8n, YOLOv10n, YOLOv11n, and MSCDNet, with MSCDNet detecting four tanks—demonstrating superior low-light detection capability compared to other models. In the fifth column, under heavy smoke interference: YOLOv8n and YOLOv10n exhibited both missed detections and false positives for the two tank targets, YOLOv11n showed missed detections with low recognition rates, and MSCDNet accurately identified one target with high confidence and zero false positives. This demonstrated MSCDNet’s superior detection capability in smoke-obscured environments compared to other models. Comparative analysis demonstrated our model’s superior detection capability in multi-scale, complex, and partially occluded environments. While maintaining better performance than other models, the system still exhibited missed detections under extreme low-light and dense smoke interference, highlighting our model’s advantages in challenging scenarios with varying scales, complex background interference, and partial occlusions.

We evaluated various module configurations for battlefield object recognition within the YOLOv11 architecture, addressing challenges such as variable scales, complex morphologies, and cluttered backgrounds. Table 3 shows performance metrics for five YOLOv11 variants. The proposed MSFM module outperformed others with 84.9% precision, surpassing the C3k2 baseline by 3.1 percentage points, and maintained a competitive recall of 65.8%. It achieved the highest detection quality, with an mAP50 of 72.5% and an mAP50-95 of 39.1%. The C3k2-Star variant showed a slight precision improvement to 82.2%, but its recall dropped significantly to 61.5%, limiting its effectiveness. The C3k2-IDWC variant struck a balance with 83.0% precision and good computational efficiency at 6.1 GFLOPS, making it the most lightweight option. The MAN module achieved 83.2% precision but struggled with recall at 62.2% and introduced higher computational costs at 8.4 GFLOPS and 3.77 M parameters. MSFM stood out by providing superior detection performance without additional computational overhead, maintaining the same GFLOPS as the baseline C3k2, with only minor parameter differences, making it the optimal choice for object recognition in resource-constrained environments due to its balanced performance and efficiency.

Table 4 presents the performance metrics of various FPN architectures, highlighting the advantages of our approach over existing methods. Traditional FPN shows significant limitations in military target detection. The Slimneck architecture exhibits suboptimal feature representation with a recall of 65.2%. BiFPN, while computationally efficient, struggles with complex targets, achieving a recall of 66.4%. MAFPN improves precision to 84.0%, but its recall drops to 65.1% and requires higher computational resources at 7.1 GFLOPS. Our architecture overcomes these limitations, achieving optimal precision of 82.4% and superior recall of 67.8%, surpassing Slimneck by 2.6%, BiFPN by 1.4%, and MAFPN by 2.7%. This improvement translates into better mean average precision metrics, with an mAP50 of 72.6% and mAP50-95 of 38.6%. Remarkably, our design maintains a moderate computational cost of 6.3 GFLOPS—comparable to BiFPN and 11.3% lower than MAFPN. This balance is achieved through the MSFM module, which enhances multi-scale feature extraction, and the CMM module, which enables advanced context-aware feature fusion. Together, these modules offer an efficient and high-performance solution for object recognition in resource-constrained environments.

### 4.5. Generalization Experiments

To assess the adaptability of our model across different domains, we evaluated its performance on two distinct datasets.

The VisDrone2019 [63] dataset serves as a comprehensive benchmark for visual object detection in aerial imagery. It includes around 10,000 images and 288 video clips, with 2.6 million annotated objects spanning 10 categories. The dataset is divided into 6471 images for training, 548 for validation, and 3190 for testing.

The BDD100K [64] dataset presents challenges from a vehicle-mounted perspective. With over 100,000 images and 10 million annotations, it covers both various weather conditions as well as day and night environments. For this dataset, we used 70,000 images for training, 10,000 for validation, and 20,000 for testing.

Comparing our model to the baseline YOLO11n, we observed consistent improvements across both datasets as shown in Table 5. On VisDrone, precision increased from 45.5% to 45.7%, a gain of 0.2%. Recall improved from 33.4% to 35.1%, an increase of 1.7%. For detection accuracy, mAP50 rose from 33.7% to 34.8%, a gain of 1.1%, and mAP50-95 improved from 19.6% to 20.1%, an increase of 0.5%. On BDD100K, precision improved from 58.8% to 61.1%, a gain of 2.3%. However, recall decreased from 41.5% to 40.0%, a reduction of 1.5%. Overall detection performance still improved, with mAP50 increasing from 42.5% to 43.6%, a gain of 1.1%, and mAP50-95 rising from 27.8% to 29.0%, an improvement of 1.2%.

**Figure 10:**
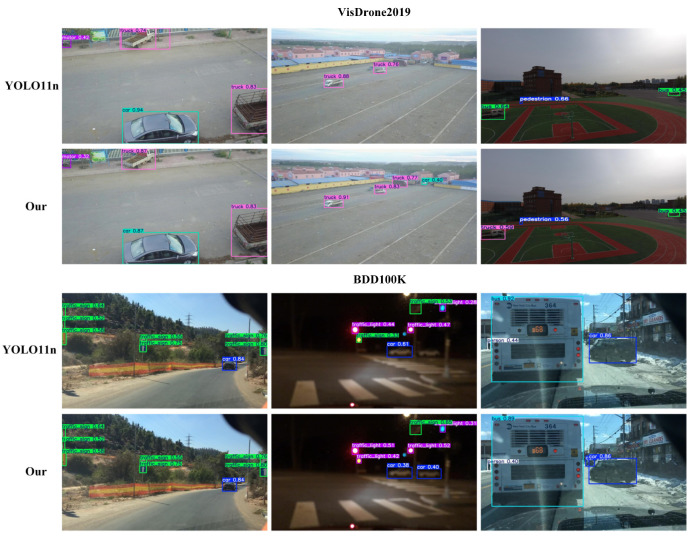
Comparison of model performance on VisDrone2019 and BDD100K before and after applying the proposed method.

These results highlight the superior detection performance of our model across different domains. Figure 10 demonstrates the improved detection effectiveness of our model before and after the enhancements, evaluated on both the VisDrone and BDD100K datasets, visually reinforcing our quantitative findings. While some confidence scores may have appeared lower in certain instances, the overall improvements demonstrated significant advantages: (1) detection of previously missed small-scale objects, particularly in complex aerial scenarios where baseline models failed completely; (2) enhanced localization accuracy with more precise bounding box placement; (3) reduction in false positive detections, leading to higher precision; (4) more stable performance across different object scales and environmental conditions. The apparent reduction in some confidence scores actually reflected improved calibration, where the model provided more realistic confidence estimates rather than overconfident predictions.

Due to resource constraints, we were unable to conduct real-time testing on edge platforms such as NVIDIA Jetson Nano or Raspberry Pi in this study. However, our current experimental setup provided substantial evidence for resource efficiency claims through well-established theoretical analysis validated by extensive literature. Our model achieved only 6.0 G FLOPs and 2.22 M parameters, representing a 14% reduction in parameters compared to YOLOv11n baseline. Recent studies have demonstrated strong correlations between theoretical metrics and practical deployment performance [65,66,67,68,69,70]. Specifically, research by Alqahtani et al. [65] shows that models with FLOPs below 10 G and parameters under 5 M consistently achieve real-time performance on edge devices while Zhang et al. [66] demonstrated that the 6–8 G FLOPs range enables efficient deployment on resource-constrained platforms. The extensive comparisons with state-of-the-art models (Table 2) demonstrated that MSCDNet consistently requires fewer computational resources while achieving superior performance. Studies on lightweight YOLO models [67,68] have established that parameter count and FLOPs serve as reliable predictors of edge device performance. The lightweight design principles embedded in our MSFM, CMM, and DEM modules are specifically engineered for resource-constrained deployment, following established guidelines for edge-optimized architectures [69]. Our approach of avoiding excessive group convolutions and element-wise operations aligns with proven strategies for edge deployment, as demonstrated in recent work on Edge-YOLO [70]. In our future work, we plan to deploy and evaluate the model on these edge devices to assess its real-time performance metrics, including inference speed, memory consumption, and power efficiency.

## 5. Conclusions

This paper has introduced MSCDNet, a lightweight architecture for target detection in resource-constrained environments. Our approach integrates three key modules: Multi-Scale Fusion for enhanced feature representation, Context Merge Module for adaptive cross-scale integration, and Detail Enhance Module for preserving critical details. Experiments demonstrate MSCDNet’s superior performance with 40.1% mAP50-95, 86.1% precision, and 68.1% recall while requiring minimal computational resources of just 2.22 M parameters and 6.0 G FLOPs. Our model consistently outperforms contemporary architectures including YOLO variants while using fewer resources. Generalization tests across VisDrone2019 and BDD100K datasets confirm its effectiveness in diverse scenarios.

Despite these achievements, limitations remain in extreme weather conditions and severe occlusion. The model also requires deployment and performance evaluation on edge devices to assess critical real-time metrics including inference speed, memory consumption, and power efficiency, followed by subsequent optimization. Future work should explore model deployment and validation testing on edge devices, cross-modal fusion for all-weather capability, adaptive computation mechanisms, self-supervised learning approaches, and tests under more challenging experimental conditions including extreme environments and night vision scenarios and hardware-aware optimizations to further enhance MSCDNet’s applicability in resource-constrained environments where detection reliability directly impacts the application value in civilian fields such as personnel search and rescue, traffic management, etc.

While this research demonstrates advances in lightweight object detection, we emphasize that the primary intention is to enhance civilian safety applications such as search and rescue operations, traffic monitoring, and disaster response. The authors advocate for the responsible deployment and ethical use of this technology in accordance with international humanitarian principles.

## Figures and Tables

**Table 1:** Ablation experimental results.

YOLO11n	MSFM	CMM	DEM	Param (M)	FLOPs (G)	P (%)	R (%)	mAP50 (%)	mAP50-95 (%)
√				2.58	6.3	81.8	66.5	71.5	38.2
√	√			2.53	6.3	84.9	65.8	72.5	39.1
√		√		2.59	6.3	82.4	67.8	72.6	38.6
√			√	2.26	6.0	83.4	67.6	72.5	39.6
√	√	√		2.54	6.3	85.5	66.7	73.7	39.5
√	√		√	2.21	6.0	84.1	67.9	73.6	38.9
√		√	√	2.26	6.0	83.7	67.0	73.2	39.6
√	√	√	√	2.22	6.0	86.1	68.1	74.7	40.1

**Table 2:** Comparison of the test results of different models.

Model	Precision (%)	Recall (%)	mAP50 (%)	mAP50-95 (%)	Flops (G)	Param (M)
SSD	76.2	58.3	63.7	31.8	31.2	27.1
DETR	82.4	61.5	67.9	35.2	95.2	44.0
TOOD	83.5	63.7	70.2	37.1	199	32.04
RTMDet-Tiny	83.2	64.3	70.5	37.3	8.03	4.87
DFINE-n	82.8	64.5	70.6	37.5	7.12	3.73
DEIM-n	83.0	64.6	70.7	37.5	7.12	3.73
YOLOv5n	85.3	64.7	70.8	37.6	5.80	2.18
YOLOv8n	83.0	64.9	71.4	37.8	8.1	3.0
YOLOv10n	83.9	63.8	70.8	38.3	8.2	2.69
YOLOv11n	81.8	66.5	71.5	38.2	6.3	2.58
Our	86.1	68.1	74.7	40.1	6.0	2.22

**Table 3:** Comparative analysis of YOLOv11 module variants for object recognition.

Model	Precision (%)	Recall (%)	mAP50 (%)	mAP50-95 (%)	Flops (G)	Param (M)
C3k2	81.8	66.5	71.5	38.2	6.3	2.58
C3k2-Star [57]	82.2	61.5	68.3	35.5	6.4	2.47
C3k2-IDWC [58]	83.0	65.0	71.4	37.3	6.1	2.39
MAN [59]	83.2	62.2	70.2	37.1	8.4	3.77
MSFM	84.9	65.8	72.5	39.1	6.3	2.53

**Table 4:** Performance comparison of various FPN architectures in object recognition.

Model	Precision (%)	Recall (%)	mAP50 (%)	mAP50-95 (%)	Flops (G)	Param (M)
Slimneck [60]	81.8	65.2	70.1	37.0	5.9	2.57
BiFPN [61]	82.0	66.4	70.9	37.6	6.3	1.92
MAFPN [62]	84.0	65.1	70.6	37.5	7.1	2.69
CMM	82.4	67.8	72.6	38.6	6.3	2.59

**Table 5:** Generalized experimental results on different datasets.

Dataset	Model	Precision (%)	Recall (%)	mAP50 (%)	mAP50-95 (%)
VisDrone	YOLO11n	45.5	33.4	33.7	19.6
	Our	45.7	35.1	34.8	20.1
BDD100K	YOLO11n	58.8	41.5	42.5	27.8
	Our	61.1	40.0	43.6	29.0

## Data Availability

The data presented in this study are available on request from the corresponding author.
